# Identification of Plasmid-Mediated Colistin Resistance in Multidrug-Resistant Gram-Negative Rods Isolated From Immunocompromised Patients

**DOI:** 10.7759/cureus.111295

**Published:** 2026-06-22

**Authors:** Yusra Wahab, Humera Javed, Shah Jahan, Farzana Khan, Kokab Jabeen

**Affiliations:** 1 Pathology and Laboratory Medicine, Faisalabad Medical University, Faisalabad, PAK; 2 Microbiology, Children's Hospital Lahore, University of Child Health Sciences, Lahore, Pakistan; 3 Immunology, University of Health Sciences, Lahore, PAK; 4 Pediatric Medicine, PAF (Pakistan Air Force) Hospital Islamabad, Islamabad, PAK; 5 Microbiology, University of Health Sciences, Lahore, PAK

**Keywords:** colistin broth disc elution (cbde), colistin resistance, gram negative rods, immunocompromised patients, mcr-1 gene

## Abstract

Introduction: Antimicrobial resistance (AMR) poses a catastrophic threat to immunocompromised patients, particularly through the spread of multidrug-resistant (MDR) Gram-negative bacteria. With the rise of carbapenem resistance, colistin has returned as a last-resort therapy. However, the emergence of the mobilized colistin resistance (*mcr*) gene threatens this final defense. This study evaluates the prevalence of phenotypic colistin resistance and the *mcr-1* gene in clinical isolates from immunocompromised patients.

Methods: This descriptive cross-sectional study was conducted over 12 months at Lahore General Hospital and the University of Health Sciences, Lahore. We collected samples of 100 MDR Gram-negative rods (GNRs) from biological specimens (urine, blood, pus, wound, tracheal secretions) of immunocompromised patients. Identification was confirmed via API 20E (Analytical Profile Index). Antibiotic susceptibility was determined using the Kirby-Bauer method. Crucially, colistin susceptibility was assessed using the CLSI-recommended colistin broth disk elution (CBDE) method. Polymerase chain reaction (PCR) was used to screen for *mcr-1* through *mcr-5* genes.

Results: The sample comprised 100 isolates, predominantly *Escherichia coli* (32%) and *Klebsiella pneumoniae* (25%). Phenotypic screening revealed a colistin resistance rate of 6% (6/100). Resistance was highest in *K. pneumoniae* (16%) and *Acinetobacter baumannii* (7.7%). Notably, phenotypic resistance was not observed in *Escherichia coli* or *Enterobacter cloacae* in this specific cohort. Molecular analysis confirmed that 100% (6/6) of the colistin-resistant isolates harbored the *mcr-1* gene. No isolates carried *mcr-2, -3, -4, *or* -5*. High resistance rates were observed against cephalosporins and carbapenems.

Conclusion: While the overall prevalence of colistin resistance (6%) is relatively low compared to carbapenems, the universal presence of *mcr-1* in resistant strains suggests a plasmid-mediated transmission route that poses a severe risk to immunocompromised populations. The CBDE method offers a reliable diagnostic alternative for resource-limited settings.

## Introduction

The global escalation of antimicrobial resistance (AMR) has rapidly outpaced the development of new therapeutic agents, creating a precarious scenario for high-risk patient populations. Immunocompromised individuals, including those with malignancies, organ transplants, or unmanaged diabetes, are particularly vulnerable to opportunistic infections caused by Gram-negative rods (GNRs) [1]. In Pakistan, the situation is compounded by high rates of self-medication, inadequate infection control in tertiary care settings, and the widespread empiric use of broad-spectrum antibiotics [2].

For decades, carbapenems were the bulwark against resistant GNRs. However, the dissemination of New Delhi metallo-beta-lactamase (NDM) and other carbapenemases has rendered these drugs increasingly ineffective [3]. Consequently, clinicians have been forced to resuscitate colistin (polymyxin E), a cationic polypeptide antibiotic previously discarded due to nephrotoxicity, as a salvage therapy for multidrug-resistant (MDR) infections caused by *Acinetobacter baumannii*, *Pseudomonas aeruginosa*, and Enterobacteriaceae [4].

Unfortunately, colistin resistance is no longer a theoretical threat. While resistance was historically attributed to chromosomal mutations (e.g., mgrB or pmrAB systems), which are vertically transmitted, the discovery of the plasmid-mediated *mcr-1* gene in 2015 fundamentally altered the risk landscape [5]. Because *mcr* genes are located on mobile genetic elements, they facilitate horizontal gene transfer across bacterial species, theoretically enabling an exponential spread of pan-drug resistance [6]. Since the initial discovery, variants *mcr-1* through *mcr-10* have been identified globally, yet local data regarding their specific prevalence in Pakistan’s immunocompromised population remain fragmented [7].

This study was designed to address two critical gaps in local clinical practice. First, we aimed to validate the colistin broth disk elution (CBDE) method as a practical alternative to standard disk diffusion (which is unreliable for polymyxins due to poor agar diffusion). Second, we sought to determine the specific prevalence of *mcr* variants (*mcr-1* to *mcr-5*) in MDR isolates from immunocompromised patients to inform local antimicrobial stewardship policies.

## Materials and methods

Study design and setting

This descriptive cross-sectional study was conducted over a 12-month period following synopsis approval from 15/06/2023 till 15/12/2023. Clinical isolates were sourced from the Microbiology Laboratory of Lahore General Hospital, while molecular analysis was performed at the Department of Microbiology, University of Health Sciences (UHS), Lahore.

Sample selection

A non-probability convenience sampling technique was used to collect 100 MDR Gram-negative bacterial isolates from different clinical specimens of immunocompromised patients admitted to different wards of Lahore General Hospital. The sample size was calculated using the single-population proportion formula, with a 95% confidence level and a 6% margin of error. The formula used for sample size calculation was $N=pqz^{2}/d^{2}$, where N represents the required sample size, p denotes the assumed proportion of colistin resistance taken as 0.88 according to Zafer et al. [8], and q was calculated as 1−p1-p1−p. The value of z was taken as 1.96, corresponding to a 95% confidence interval, while d represented the margin of error, taken as 7%. A non-probability convenience sampling technique was used for the selection of study participants. Only one sample was collected from each patient. No repetition of the sample was made from the same patient.

Inclusion Criteria

Isolates from patients with confirmed immunocompromised status (based on clinical history of diabetes, chemotherapy, or immunosuppressive therapy) from specimens, including urine, blood, pus, wound swabs, and tracheal secretions.

Exclusion Criteria

Repeat isolates from the same patient (to prevent data duplication) and isolates that did not display multidrug resistance phenotypic traits initially.

Primary Outcome

To determine plasmid-mediated colistin resistance among immunocompromised patients using the disk elution method and polymerase chain reaction (PCR).

Secondary Outcomes

To assess colistin resistance in MDR GNRs isolated from immunocompromised patients by using the disk elution method. To evaluate the antibiotic susceptibility patterns of the isolated organisms. To identify the presence of *mcr-1* to *mcr-5* resistance genes in the bacterial isolates.

Bacterial identification

Samples were cultured on Blood Agar, MacConkey Agar, and CLED Agar. Identification was performed using Gram staining and standard biochemical profiles (catalase, citrate, indole, motility, triple sugar iron, urease). Confirmation was achieved using the API 20E (Analytical Profile Index) system (BioMérieux, Marcy-l'Étoile, France).

Antimicrobial susceptibility testing (AST)

Susceptibility testing was performed using the Kirby-Bauer disk diffusion method on Mueller-Hinton Agar (MHA) per Clinical and Laboratory Standards Institute (CLSI) 2022 guidelines [8]. The antibiotic panel included ampicillin, amoxicillin/clavulanate, aztreonam, cefepime, cefotaxime, cefoperazone, ceftazidime, imipenem, meropenem, clindamycin, gentamicin, amikacin, and co-trimoxazole.

Colistin susceptibility: Broth disk elution method (CBDE)

Recognizing the limitations of disk diffusion for colistin due to its poor diffusion characteristics in agar media, we employed the CBDE method, which is recommended by the CLSI as a reliable alternative for colistin susceptibility testing [8]. The CBDE method was performed using four 10 mL cation-adjusted Mueller-Hinton broth (CA-MHB; Remel, Lenexa, KS) tubes per isolate.

Elution

Colistin disks (10 µg) were added to tubes to create final concentrations of 0 (control), 1, 2, and 4 µg/mL.

Inoculation

Tubes were inoculated with 50 µL of a standardized 0.5 McFarland bacterial suspension and incubated at 35°C for 16-20 hours.

Interpretation

Growth in the 4 µg/mL tube indicated resistance (minimum inhibitory concentration (MIC) ≥ 4 µg/mL) [9]. 

Molecular Detection of mcr Genes

DNA was extracted using the heat extraction method. A bacterial suspension of 2-3 colonies was prepared in 200 µL of Tris-EDTA (TE) buffer, boiled for 10 minutes, and then centrifuged. The supernatant was used as the DNA template.

Multiplex PCR was optimized for the detection of *mcr-1*, *mcr-2*, *mcr-3*, *mcr-4*, and *mcr-5* genes using specific primers and DreamTaq Master Mix (Thermo Fisher Scientific, Waltham, MA, USA). Amplification was performed in a final reaction volume of 25 µL using a thermal cycler (Bio-Rad, Hercules, CA, USA). The PCR conditions included an initial denaturation at 95°C for 3 minutes, followed by 30 cycles of denaturation at 94°C, annealing at 58°C, and extension at 72°C.

PCR products were visualized on a 1.5% agarose gel using SYBR™ Safe DNA gel stain (Invitrogen, Carlsbad, CA, USA) and 6× loading dye.

Intrinsic resistance, as defined by CLSI guidelines, is inherent or innate (not acquired) AMR naturally present in the wild-type antimicrobial susceptibility patterns of all or almost all representatives of a bacterial species.

All statistical analyses were performed using IBM SPSS software version 26 (IBM Corp., Armonk, NY, USA). The data were recorded and analyzed using IBM SPSS software (version 26). Mean and standard deviation (SD) were calculated for quantitative variables such as MICs and size of zones of inhibition. Frequencies were reported for qualitative variables such as variants, sensitivity, and resistance. The data related to the *mcr-1* gene (colistin resistance) were presented in graphical form using SPSS software.

## Results

The study included 100 clinical isolates. A gender disparity was observed, with 59% of samples originating from female patients and 41% from male patients. Urine was the most frequent source of MDR isolates (32%), followed by pus (29%) and blood (16%), as mentioned in Table 1.

**Table 1:** Frequency distribution of clinical specimens and patient gender (n=100)

Characteristics	Total No. of Samples	Percentage
Clinical samples		
Urine	32	32
Pus	29	29
Blood	16	16
Wound	18	18
Tracheal	5	5
Gender		
Male	41	41
Female	59	59

Microbiological profile

**Figure 1:**
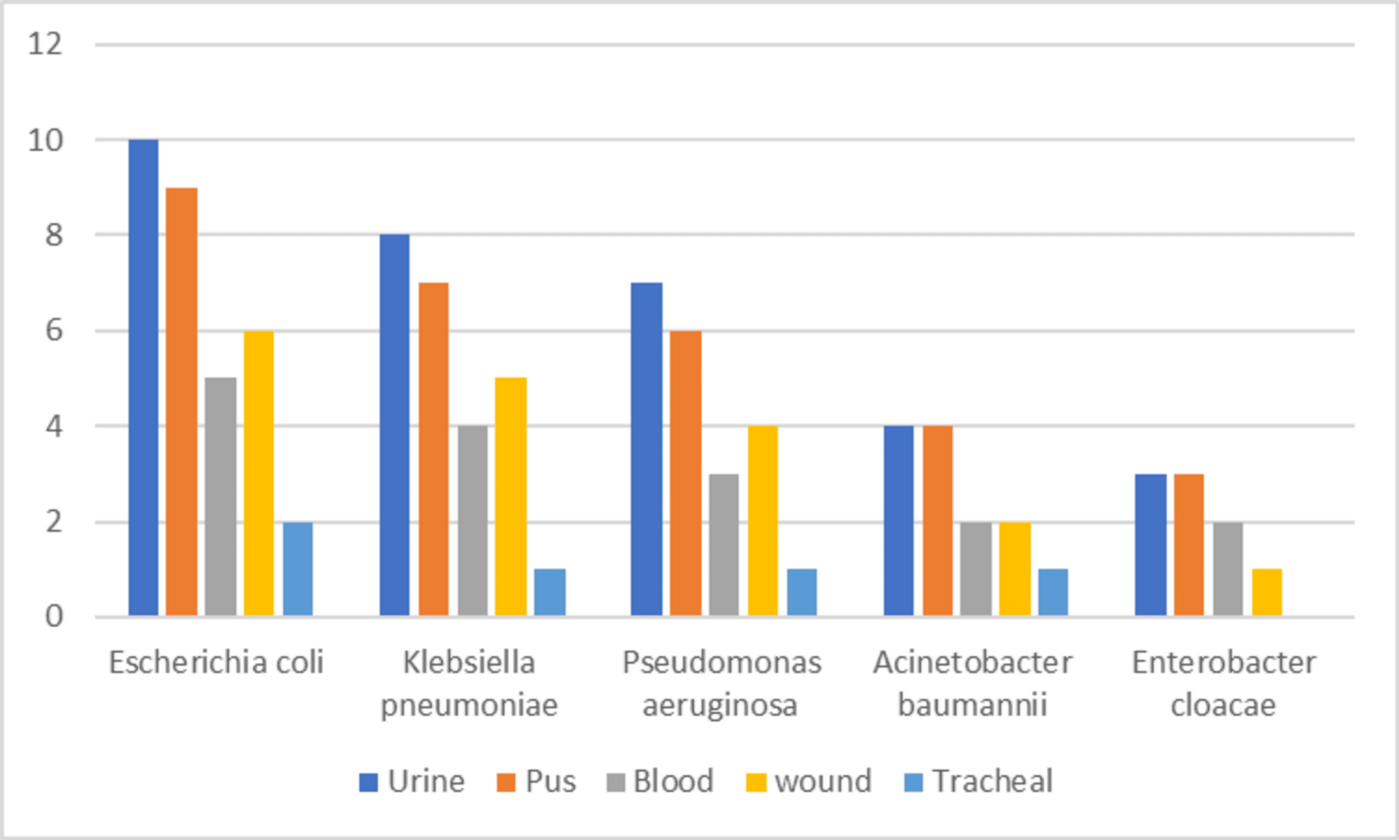
Distribution of Gram-negative rods isolated from clinical specimens The bar chart displays the frequency of isolated pathogens. *Escherichia coli *was the most prevalent organism (32%), followed by *Klebsiella pneumoniae* (25%) and *Pseudomonas aeruginosa* (21%). The total number of isolates was 100. The y-axis shows the number of organisms in each specimen. GNR: Gram-negative rods.

*Escherichia coli* was the predominant pathogen (32%), followed by *Klebsiella pneumoniae* (25%) and *P. aeruginosa* (21%). *Acinetobacter baumannii*, a critical pathogen in hospital-acquired infections, accounted for 13% of the total burden (Figure 1).

Antibiotic resistance profile

The susceptibility profile (Table 2) of the isolated GNRs revealed alarming rates of resistance across multiple antibiotic classes in immunocompromised patients. Given that urine was the primary clinical specimen, accounting for 32% of all samples, the resistance patterns of common urinary pathogens like *Escherichia coli* and *K. pneumoniae* are of particular clinical concern. *Escherichia coli*, the most frequent isolate in urine, showed a 28.1% resistance rate to both imipenem and meropenem, a 9.3% resistance to amikacin, and a significant 40.6% resistance to the urinary-relevant antibiotic trimethoprim/sulfamethoxazole. Meanwhile, *K. pneumoniae* exhibited extreme resistance, with 100% resistance to cefixime, ceftriaxone, and cefoperazone. This pathogen also demonstrated high levels of carbapenem resistance (80% for meropenem and 68% for imipenem) and an 84% resistance rate to amikacin.

**Table 2:** Antibiotic resistance profile of multidrug-resistant Gram-negative isolates according to CLSI 2022 guidelines Values represent the number and percentage of resistant isolates for each organism. Percentages were calculated using the total number of isolates for each bacterial species as the denominator: *Escherichia coli* (n=32), *Klebsiella pneumoniae* (n=25), *Pseudomonas aeruginosa* (n=21), *Acinetobacter baumannii* (n=13), and *Enterobacter cloacae* (n=9). Carbapenem resistance was determined based on resistance to imipenem and/or meropenem. The total resistant isolate count represents the sum of resistant isolates among organisms tested for that antibiotic only. Intrinsically resistant organisms and isolates not tested were excluded from the calculation. NT: not tested; NA: not applicable; IR: intrinsic resistance (as per CLSI guidelines); CLSI: Clinical and Laboratory Standards Institute.

Antibiotics	*Escherichia coli* (n=32)	*Klebsiella pneumoniae* (n=25)	*Pseudomonas aeruginosa* (n=21)	*Acinetobacter baumannii* (n=13)	*Enterobacter cloacae* (n=9)	Total Resistant Isolates
Ampicillin	15 (46.9%)	NT	IR	NT	NT	15
Co-amoxiclav	23 (71.9%)	NT	IR	NT	NT	23
Aztreonam	5 (15.6%)	18 (72%)	20 (95.2%)	NT	4 (44.4%)	47
Cefuroxime	2 (6.3%)	0 (0%)	IR	NT	NT	2
Cefixime	2 (6.3%)	1 (4%)	IR	NT	NT	3
Ceftriaxone	2 (6.3%)	1 (4%)	IR	NT	NT	3
Cefepime	14 (43.8%)	15 (60%)	19 (90.4%)	4 (30.8%)	1 (11.1%)	53
Cefotaxime	19 (59.4%)	18 (72%)	IR	NT	6 (66.7%)	43
Cefoperazone	8 (25%)	25 (100%)	21 (100%)	6 (46.2%)	2 (22.2%)	62
Ceftazidime	19 (59.4%)	25 (100%)	19 (90.4%)	NT	4 (44.4%)	67
Imipenem	9 (28.1%)	17 (68%)	19 (90.4%)	7 (53.8%)	9 (100%)	61
Meropenem	9 (28.1%)	20 (80%)	6 (28.6%)	8 (61.5%)	1 (11.1%)	44
Clindamycin	6 (18.8%)	20 (80%)	21 (100%)	8 (61.5%)	7 (77.8%)	62
Colistin	2 (6.3%)	1 (4%)	1 (4.8%)	1 (7.7%)	1 (11.1%)	6
Gentamicin	4 (12.5%)	15 (60%)	6 (28.6%)	6 (46.2%)	5 (55.6%)	36
Amikacin	3 (9.4%)	21 (84%)	10 (47.6%)	13 (100%)	9 (100%)	56
Amoxycillin/clavulanic acid	19 (59.4%)	11 (44%)	21 (100%)	8 (61.5%)	3 (33.3%)	62
Trimethoprim/sulphamethoxazole	13 (40.6%)	9 (36%)	15 (71.4%)	7 (53.8%)	4 (44.4%)	48
Chloramphenicol	NT	NT	NT	NT	NT	NA

The study further highlights severe resistance among pathogens requiring specialized therapeutic approaches, such as *A. baumannii* and *P. aeruginosa*. For *A. baumannii*, resistance to carbapenems was critical, recorded at 61.5% for meropenem and 53.8% for imipenem, while the pathogen showed absolute (100%) resistance to amikacin. Expanded testing for *A. baumannii* also revealed resistance to gentamicin (46.1%), cefoperazone (46.1%), and cefepime (30.7%). *Pseudomonas aeruginosa* exhibited a similarly challenging profile, with 95.2% resistance to aztreonam and 90.4% resistance to imipenem, although resistance to meropenem was notably lower at 28%. Further anti-pseudomonal testing showed 100% resistance to cefoperazone and 90.4% resistance to both cefepime and ceftazidime, alongside 47.6% resistance to amikacin and 28% to gentamicin.

Colistin resistance and *mcr* gene prevalence

**Figure 2:**
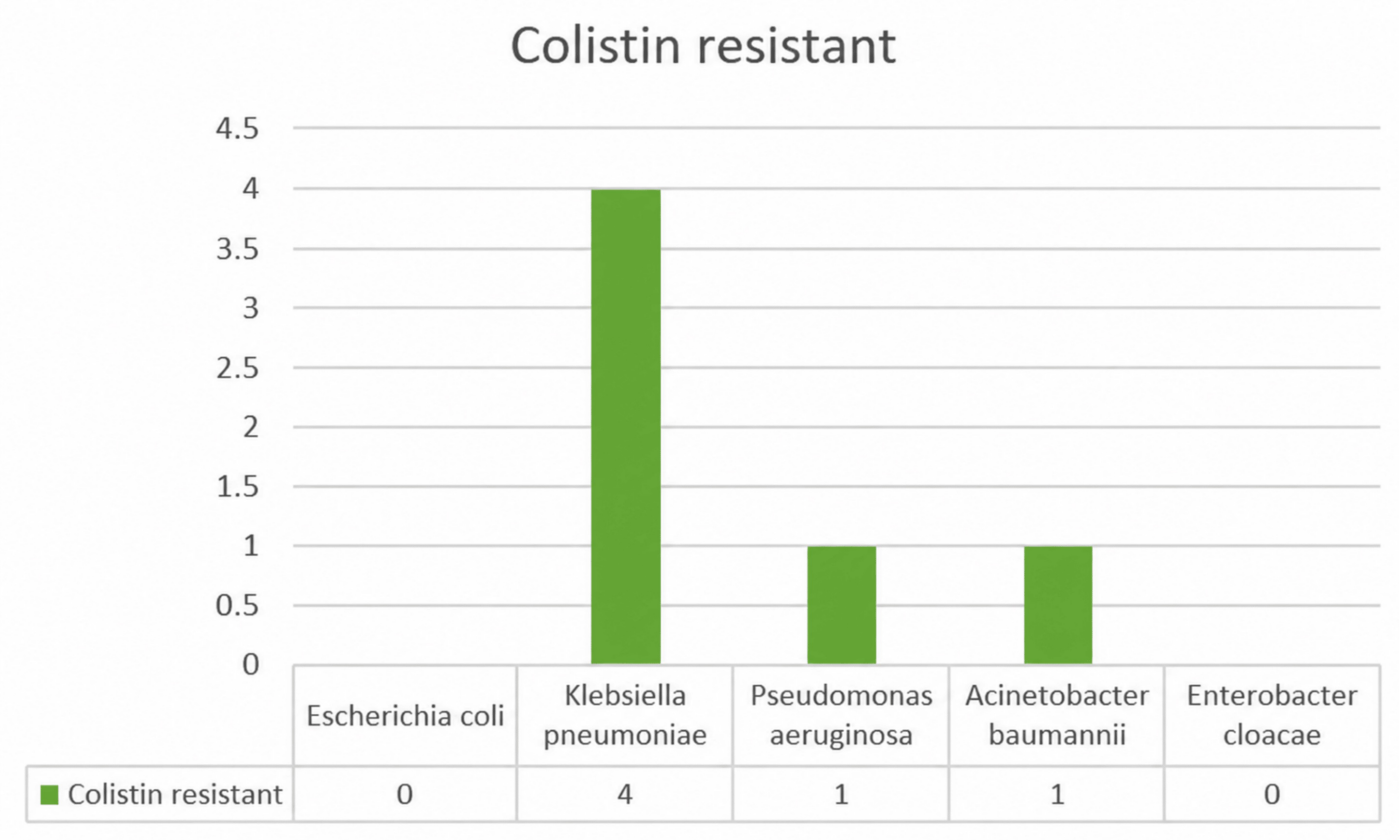
Distribution of phenotypically colistin-resistant Gram-negative bacterial isolates detected by the CBDE method Y-axis label: Number of colistin-resistant isolates. X-axis label: Bacterial species. CBDE: colistin broth disk elution.

Phenotypic testing via CBDE identified six isolates (6%) as colistin-resistant. The distribution of these resistant isolates was uneven across species. *Klebsiella pneumoniae* exhibited the highest resistance rate (16%, n=4), followed by *A. baumannii* (7.7%, n=1) and *P. aeruginosa* (4.8%, n=1). Interestingly, despite being the most common isolate, *Escherichia coli* showed 0% phenotypic resistance to colistin in this study (Figure 2).

Molecular analysis

PCR analysis was conducted on the six colistin-resistant isolates. All six (100%) tested positive for the *mcr-1* gene. No isolates harbored *mcr-2*, *mcr-3*, *mcr-4*, or *mcr-5* (Table 3).

**Table 3:** Distribution of mcr-1 to mcr-5 genes among colistin-resistant isolates Percentages were calculated using the total number of colistin-resistant isolates tested by PCR (n=6). All six isolates were positive for *mcr-1*, while no isolates carried *mcr-2*, *mcr-3*, *mcr-4*, or *mcr-5*. mcr: mobilized colistin resistance gene.

Isolate	*mcr-1* (n=6)	*mcr-2, -3, -4, -5* (n=0)
Escherichia coli	0 (0%)	0
Klebsiella pneumoniae	4 (66.7%)	0
Pseudomonas aeruginosa	1 (16.7%)	0
Acinetobacter baumannii	1 (16.7%)	0
Enterobacter cloacae	0 (0%)	0

## Discussion

This study aimed to investigate plasmid-mediated colistin resistance among MDR GNRs isolated from immunocompromised patients. The emergence of colistin resistance has become a major global healthcare concern because colistin is considered one of the last-resort antibiotics for treating MDR bacterial infections. The increasing prevalence of colistin-resistant organisms threatens the effectiveness of current therapeutic options and significantly affects patient outcomes, particularly among immunocompromised individuals who are highly vulnerable to severe infections [10,11]. Therefore, understanding the prevalence, resistance patterns, and molecular mechanisms associated with colistin resistance is essential for improving infection control strategies and antimicrobial stewardship practices.

In this study, 100 clinical samples were collected from immunocompromised patients. The isolates were obtained from different clinical specimens, including urine, blood, pus, wound swabs, and tracheal secretions, reflecting the diversity of infections encountered in hospital settings. Female patients constituted the majority of participants (59%), while male patients represented 41% of the study population. Among the isolated pathogens, *Escherichia coli* was the most frequently identified organism, followed by *K. pneumoniae*, *P. aeruginosa*, *A. baumannii*, and *Enterobacter cloacae*. The predominance of *Escherichia coli* in urinary and bloodstream infections is consistent with previously reported findings highlighting its important role in hospital-acquired infections.

Antimicrobial susceptibility testing was performed using the Kirby-Bauer disc diffusion method and MIC determination for colistin resistance. The findings demonstrated alarming levels of AMR among Gram-negative isolates. *Escherichia coli* isolates exhibited high resistance to ampicillin, co-amoxiclav, cefotaxime, and amoxicillin/clavulanic acid. Similarly, *K. pneumoniae* showed extensive resistance to multiple antibiotics, including cefoperazone, ceftazidime, amikacin, meropenem, and carbapenems. *Pseudomonas aeruginosa* demonstrated high resistance rates against aztreonam, cefepime, ceftazidime, and cefoperazone, while *A. baumannii* and *Enterobacter cloacae* displayed substantial resistance to carbapenems and aminoglycosides. These findings are in agreement with previous studies conducted in Pakistan and other developing countries, where increasing multidrug resistance among Gram-negative bacteria has become a significant clinical challenge [12,13].

One of the most important findings of this study was the detection of colistin resistance among MDR Gram-negative isolates. Out of the total isolates tested, six isolates were phenotypically resistant to colistin. Molecular analysis further revealed that all six colistin-resistant isolates carried the plasmid-mediated *mcr-1* gene, while no isolates were positive for *mcr-2*, *mcr-3*, *mcr-4*, or *mcr-5*. Among the *mcr-1*-positive isolates, four were identified as *K. pneumoniae*, one as *P. aeruginosa*, and one as *A. baumannii*. The identification of plasmid-mediated resistance is particularly concerning because plasmids facilitate horizontal gene transfer between bacterial species, thereby accelerating the spread of resistance within healthcare settings [14].

The presence of the *mcr-1* gene in clinical isolates observed in this study is consistent with previous reports from Pakistan and other countries. Hameed et al. [15] first reported plasmid-mediated *mcr-1* in *A. baumannii* and *P. aeruginosa* in Pakistan, while Bilal et al. [16] documented the presence of *mcr-1* in ESBL (extended-spectrum beta-lactamase)-producing *K. pneumoniae*. These findings collectively indicate the growing dissemination of plasmid-mediated colistin resistance in the region. The spread of *mcr* genes poses a major threat because it compromises the effectiveness of colistin, which is often reserved for critically ill patients with limited treatment options.

The emergence of MDR and colistin-resistant pathogens among immunocompromised patients has serious clinical implications. These patients frequently undergo invasive procedures, prolonged hospitalization, chemotherapy, organ transplantation, or immunosuppressive therapy, all of which increase their susceptibility to opportunistic infections. Additionally, repeated exposure to broad-spectrum antibiotics contributes to the selective pressure responsible for the development and spread of resistant organisms [17]. Consequently, infections caused by MDR Gram-negative bacteria are associated with increased morbidity, mortality, prolonged hospital stays, and higher healthcare costs.

The findings of this study further emphasize the urgent need for effective antimicrobial stewardship programs and infection prevention strategies. Inappropriate and excessive antibiotic use remains one of the primary drivers of AMR worldwide [18]. In many developing countries, including Pakistan, the irrational use of antibiotics, over-the-counter availability, self-medication, and inadequate infection control practices contribute significantly to the rapid emergence of resistant pathogens [19]. Therefore, strengthening antibiotic stewardship policies, promoting rational antibiotic prescribing, and ensuring compliance with infection control measures are essential steps to reduce the burden of AMR.

Healthcare-associated infections caused by MDR organisms can be minimized through strict infection prevention measures such as hand hygiene, environmental disinfection, surveillance systems, isolation protocols, and antimicrobial monitoring programs [20]. Early identification and molecular characterization of resistant strains are also important for timely clinical management and outbreak control. The detection of plasmid-mediated colistin resistance genes provides valuable epidemiological information that can support hospital surveillance programs and guide therapeutic decision-making.

Another important aspect highlighted in this study is the growing global concern regarding AMR. AMR is now recognized as one of the most serious public health threats worldwide. The rapid dissemination of resistance genes across bacterial populations limits available treatment options and threatens the success of modern medical procedures such as surgery, transplantation, and cancer chemotherapy [21]. The World Health Organization has repeatedly emphasized the urgent need for coordinated global action to combat AMR through surveillance, research, antibiotic stewardship, and public awareness campaigns.

Despite the significant findings, this study has certain limitations. First, the study was conducted at a single center with a relatively limited sample size, which may restrict the generalizability of the findings to larger populations. Second, the study focused specifically on immunocompromised patients; therefore, the resistance patterns observed may differ from those seen in immunocompetent individuals. Third, only selected *mcr* genes were investigated, while other resistance mechanisms associated with colistin resistance may also be present. Future multicenter studies with larger sample sizes are therefore needed to provide a broader understanding of the epidemiology of colistin resistance in Pakistan and other regions.

Further research should also focus on exploring the molecular mechanisms underlying colistin resistance and the role of mobile genetic elements in the transmission of resistance genes. Detailed genomic studies may help identify additional resistance determinants and provide insights into the evolution and dissemination of MDR pathogens. Comparative studies between immunocompromised and immunocompetent populations may further clarify the influence of host factors on the development of AMR.

## Conclusions

Antimicrobial resistance continues to pose a significant global health challenge, particularly with the increasing emergence of MDR Gram-negative bacteria. This study identified the presence of plasmid-mediated colistin resistance among isolates obtained from immunocompromised patients, highlighting a concerning trend in AMR.

The detection of plasmid-borne resistance genes is particularly alarming because these genetic elements facilitate the rapid spread of resistance among bacterial populations. Given that colistin is often used as a last-resort antibiotic, the emergence of resistance may significantly limit available treatment options.

Strengthening antimicrobial stewardship programs, improving surveillance systems, and promoting responsible antibiotic use are critical strategies to control the spread of resistant pathogens. Continued research on resistance mechanisms and effective infection control practices will be essential for addressing the growing threat of AMR and protecting vulnerable patient populations.
